# Supplementary material for: Between darkness and light: spring habitats provide new perspectives for modern researchers on groundwater biology
Source: PeerJ. 2021 Jul 26;9:e11711. doi: 10.7717/peerj.11711 (PMC8320523; doi:10.7717/peerj.11711)
Supplement: Supplemental Information 2 [file peerj-09-11711-s002.docx]

**References list for the research key “Groundwater fauna”**

Abrams, K. M., M. T. Guzik, S. J. B. Cooper, W. F. Humphreys, R. A. King, J. L. Cho & A. D. Austin, 2012. What lies beneath: Molecular phylogenetics and ancestral state reconstruction of the ancient subterranean Australian Parabathynellidae (Syncarida, Crustacea). Molecular Phylogenetics and Evolution 64: 130-144.

Abrams, K. M., R. A. King, M. T. Guzik, S. J. B. Cooper & A. D. Austin, 2013. Molecular phylogenetic, morphological and biogeographic evidence for a new genus of parabathynellid crustaceans (Syncarida : Bathynellacea) from groundwater in an ancient southern Australian landscape. Invertebrate Systematics 27: 146-172.

Achurra, A. & P. Rodriguez, 2008. Biodiversity of groundwater oligochaetes from a karst unit in northern Iberian Peninsula: ranking subterranean sites for conservation management. Hydrobiologia 605: 159-171.

Achurra, A. & P. Rodriguez, 2016. Syntopy in subterranean fauna: Trophic specialisation in two new species of Rhyacodrilus Bretscher, 1901 (Annelida, Clitellata, Rhyacodrilinae). Zoologischer Anzeiger 261: 1-11.

Achurra, A., P. Rodriguez & C. Erseus, 2015a. Pseudo-cryptic speciation in the subterranean medium: A new species of Stylodrilus Claparede, 1862, with a revision of the status of Bichaeta Bretscher, 1900 (Annelida, Clitellata, Lumbriculidae). Zoologischer Anzeiger 257: 71-86.

Achurra, A., P. Rodriguez & T. B. Reynoldson, 2015b. Is the Cantabrian region of northern Spain a biodiversity hotspot for obligate groundwater fauna? The case of oligochaetes (Annelida, Clitellata). Hydrobiologia 745: 151-166.

Allford, A., S. J. B. Cooper, W. F. Humphreys & A. D. Austin, 2008. Diversity and distribution of groundwater fauna in a calcrete aquifer: does sampling method influence the story? Invertebrate Systematics 22: 127-138.

Apostolov, A., 2005. Stygobitic harpacticoid copepods of France. 7. Description of two new copepods of the genus Parastenocaris kessler, 1913. Crustaceana 78: 95-111.

Asmyhr, M. G. & S. J. B. Cooper, 2012. Difficulties barcoding in the dark: the case of crustacean stygofauna from eastern Australia. Invertebrate Systematics 26: 583-591.

Asmyhr, M. G., G. Hose, P. Graham & A. J. Stow, 2014a. Fine-scale genetics of subterranean syncarids. Freshwater Biology 59: 1-11.

Asmyhr, M. G., S. Linke, G. Hose & D. A. Nipperess, 2014b. Systematic Conservation Planning for Groundwater Ecosystems Using Phylogenetic Diversity. Plos One 9.

Asmyhr, M. G., A. J. Stow & G. Hose, 2012. The first set of microsatellite markers developed for the ancient Parabathynellidae (Syncarida, Malacostraca) and their utility for other groundwater fauna. Conservation Genetics Resources 4: 587-589.

Avramov, M., T. M. Rock, G. Pfister, K. W. Schramm, S. I. Schmidt & C. Griebler, 2013a. Catecholamine levels in groundwater and stream amphipods and their response to temperature stress. General and Comparative Endocrinology 194: 110-117.

Avramov, M., S. I. Schmidt & C. Griebler, 2013b. A new bioassay for the ecotoxicological testing of VOCs on groundwater invertebrates and the effects of toluene on Niphargus inopinatus. Aquatic Toxicology 130: 1-8.

Baltanas, A., T. Namiotko & D. L. Danielopol, 2000. Biogeography and disparity within the genus Cryptocandona (Crustacea, Ostracoda). Vie Et Milieu-Life and Environment 50: 297-310.

Baratti, M., M. Y. Khebiza & G. Messana, 2004. Microevolutionary processes in the stygobitic genus Typhlocirolana (Isopoda Flabellifera Cirolanidae) as inferred by partial 12S and 16S rDNA sequences. Journal of Zoological Systematics and Evolutionary Research 42: 27-32.

Bednar, J. P., M. Trobej, M. Schagerl & J. Waringer, 2017. Which factors shape macrozoobenthic communities in tufa springs? Results from Austrian meteogene travertine-depositing sites. Hydrobiologia 799: 293-307.

Beladjal, L., J. Mertens & H. J. Dumont, 1992. A Simple Basket Trap for Estimating Relative Abundances of Some Components of Hyporheic Faunas - Application to the Cladocera. Stygologia 7: 193-195.

Berkhoff, S. E., J. Bork & H. J. Hahn, 2009. Groundwater fauna as an indicator of surface water-groundwater interactions at a riverbank filtration site. Grundwasser 14: 3-20.

Berrady, I., K. Essafi & J. Mathieu, 2000. Comparative physico-chemical and faunal studies of two thermal springbrooks near Sidi Harazem (Morocco). Annales De Limnologie-International Journal of Limnology 36: 261-274.

Bis, B., A. Zdanowicz & M. Zalewski, 2000. Effects of catchment properties on hydrochemistry, habitat complexity and invertebrate community structure in a lowland river. Hydrobiologia 422: 369-387.

Bork, J., S. E. Berkhoff, S. Bork & H. J. Hahn, 2009. Using subsurface metazoan fauna to indicate groundwater-surface water interactions in the Nakdong River floodplain, South Korea. Hydrogeology Journal 17: 61-75.

Bork, J., S. Bork, S. E. Berkhoff & H. J. Hahn, 2008. Testing unbaited stygofauna traps for sampling performance. Limnologica 38: 105-115.

Bottazzi, E., M. C. Bruno, V. Pieri, A. Di Sabatino, L. Silveri, M. Carolli & G. Rossetti, 2011. Spatial and seasonal distribution of invertebrates in Northern Apennine rheocrene springs. Journal of Limnology 70: 77-92.

Boughrous, A. A., M. Boulanouar, M. Yacoubi & N. Coineau, 2007a. The first Microcharon (Crustacea, Isopoda, Microparasellidae) from the Moroccan North Saharan Platform. Phylogeny, origin and palaeobiogeography. Contributions to Zoology 76: 21-34.

Boughrous, A. A., M. Y. Khebiza, M. Boulanouar, C. Boutin & G. Messana, 2007b. Groundwater quality in two arid areas of Morocco: Impact of pollution on biodiversity and paleogeographic implications. Environmental Technology 28: 1299-1315.

Boukhoubza, F., A. A. Boughrous, M. Yacoubi-Khebiza, A. Jail, L. Hassani, L. L. Idrissi & A. Nejmeddine, 2008. Impact of olive oil wastewater on the physicochemical and biological quality of groundwater in the Haouz plain, South of Marrakesh (Morocco). Environmental Technology 29: 959-974.

Boulton, A. J., M. J. Dole-Olivier & P. Marmonier, 2004. Effects of sample volume and taxonomic resolution on assessment of hyporheic assemblage composition sampled using a Bou-Rouch pump. Archiv Fur Hydrobiologie 159: 327-355.

Boutin, C., 1994. Stygobiology and Historical Geology - the Age of Fuerteventura (Canary-Island), as Inferred from Its Present Stygofauna. Bulletin De La Societe Geologique De France 165: 273-285.

Bozkurt, A., 2019. Investigation of zooplankton fauna in water wells of Kuyubeli village (Adana, Turkey) with the first record of the genus Speocyclops Kiefer, 1937 (Copepoda, Cyclopoida, Cyclopidae) for Turkish inland waters. Turkish Journal of Zoology 43: 142-145.

Bradford, T., M. Adams, W. F. Humphreys, A. D. Austin & S. J. B. Cooper, 2010. DNA barcoding of stygofauna uncovers cryptic amphipod diversity in a calcrete aquifer in Western Australia's arid zone. Molecular Ecology Resources 10: 41-50.

Bradford, T. M., M. Adams, M. T. Guzik, W. F. Humphreys, A. D. Austin & S. J. B. Cooper, 2013. Patterns of population genetic variation in sympatric chiltoniid amphipods within a calcrete aquifer reveal a dynamic subterranean environment. Heredity 111: 77-85.

Brancelj, A., 2015. Two new stygobiotic copepod species from the Tibesti area (Northern Chad) and a re-description of Pilocamptus schroederi (van Douwe, 1915). Zootaxa 3994: 531-555.

Brancelj, A., C. Boonyanusith, S. Watiroyram & L. O. Sanoamuang, 2013. The groundwater-dwelling fauna of South East Asia. Journal of Limnology 72: 327-343.

Brancelj, A. & H. J. Dumont, 2007. A review of the diversity, adaptations and groundwater colonization pathways in Cladocera and Calanoida (Crustacea), two rare and contrasting groups of stygobionts. Fundamental and Applied Limnology 168: 3-17.

Brancelj, A., U. Zibrat & B. Jamnik, 2016. Differences between groundwater fauna in shallow and in deep intergranular aquifers as an indication of different characteristics of habitats and hydraulic connections. Journal of Limnology 75: 248-261.

Brown, L., T. Finston, G. Humphreys, S. Eberhard & A. Pinder, 2015. Groundwater oligochaetes show complex genetic patterns of distribution in the Pilbara region of Western Australia. Invertebrate Systematics 29: 405-420.

Bruce, N. L., 2008. New species and a new genus of Cirolanidae (Isopoda : Cymothoida : Crustacea) from groundwater in calcretes in the Pilbarra, northern Western Australia. Zootaxa: 51-64.

Brunke, M. & T. Gonser, 1999. Hyporheic invertebrates-the clinal nature of interstitial communities structured by hydrological exchange and environmental gradients. Journal of the North American Benthological Society 18: 344-362.

Bruno, M. C., J. W. Reid & S. A. Perry, 2005. A list and identification key for the freshwater, free-living copepods of Florida (USA). Journal of Crustacean Biology 25: 384-400.

Bruyere, E., M. J. Turquin & J. L. Reygrobellet, 1993. Biological Tracing - a New Approach for Groundwater Biotopes. Comptes Rendus De L Academie Des Sciences Serie Ii 317: 479-485.

Buhay, J. E. & K. A. Crandall, 2005. Subterranean phylogeography of freshwater crayfishes shows extensive gene flow and surprisingly large population sizes. Molecular Ecology 14: 4259-4273.

Camacho, A. I., B. A. Dorda, B. S. Chillon & I. Rey, 2017. The collection of Bathynellacea specimens of MNCN (CSIC) Madrid: microscope slices and DNA extract. Zookeys: 31-63.

Camacho, A. I., B. A. Dorda & I. Rey, 2012. Undisclosed Taxonomic Diversity of Bathynellacea (Malacostraca: Syncarida) in the Iberian Peninsula Revealed by Molecular Data. Journal of Crustacean Biology 32: 816-826.

Camacho, A. I., B. A. Dorda & I. Rey, 2013. Old and new taxonomic tools: description of a new genus and two new species of Bathynellidae from Spain with morphological and molecular characters. Journal of Natural History 47: 1393-1420.

Camacho, A. I., B. A. Dorda & I. Rey, 2014. Iberian Peninsula and Balearic Island Bathynellacea (Crustacea, Syncarida) database. Zookeys: 1-20.

Camacho, A. I. & P. Hancock, 2011. First record of Syncarida from Queensland, Australia, with description of two new species of Notobathynella Schminke, 1973 (Crustacea, Bathynellacea, Parabathynellidae). Journal of Natural History 45: 113-135.

Camacho, A. I. & P. Hancock, 2012. Two new species of the genus Chilibathynella Noodt, 1963 and Onychobathynella bifurcata gen. et sp nov (Crustacea: Syncarida: Parabathynellidae) from New South Wales, Australia. Journal of Natural History 46: 145-173.

Camacho, A. I., B. Hutchins, B. F. Schwartz, B. A. Dorda, A. Casado & I. Rey, 2018a. Description of a new genus and species of Bathynellidae (Crustacea: Bathynellacea) from Texas based on morphological and molecular characters. Journal of Natural History 52: 29-51.

Camacho, A. I., P. Mas-Peinado, B. A. Dorda, A. Casado, A. Brancelj, L. R. F. D. Knight, B. Hutchins, C. Bou, G. Perina & I. Rey, 2018b. Molecular tools unveil an underestimated diversity in a stygofauna family: a preliminary world phylogeny and an updated morphology of Bathynellidae (Crustacea: Bathynellacea). Zoological Journal of the Linnean Society 183: 70-96.

Camacho, A. I., P. Mas-Peinado, S. Watiroyram, A. Brancelj, E. Bandari, B. A. Dorda, A. Casado & I. Rey, 2018c. Molecular phylogeny of Parabathynellidae (Crustacea, Bathynellacea), and three new species from Thai caves. Contributions to Zoology 87: 227-260.

Camacho, A. I., R. L. Newell, Z. Crete, B. A. Dorda, A. Casado & I. Rey, 2016. Northernmost discovery of Bathynellacea (Syncarida: Bathynellidae) with description of a new species of Pacificabathynella from Alaska (USA). Journal of Natural History 50: 583-602.

Camacho, A. I., R. L. Newell & B. Reid, 2009. New records of Bathynellacea (Syncarida, Bathynellidae) in North America: three new species of the genus Pacificabathynella from Montana, USA. Journal of Natural History 43: 1805-1834.

Camacho, A. I., T. Torres, C. J. Puch, J. E. Ortiz & A. G. Valdecasas, 2006. Small-scale biogeographical patterns in some groundwater Crustacea, the syncarid, Parabathynellidae. Biodiversity and Conservation 15: 3527-3541.

Chatelliers, M. C. D., J. Juget, M. Lafont & P. Martin, 2009. Subterranean aquatic Oligochaeta. Freshwater Biology 54: 678-690.

Christian, E. & C. Spotl, 2010. Karst geology and cave fauna of Austria: a concise review. International Journal of Speleology 39: 71-90.

Claret, C., P. Marmonier, M. J. Dole-Olivier & E. Castella, 1999a. Effects of management works on the interstitial fauna of floodplain aquatic systems (River Rhone, France). Biodiversity and Conservation 8: 1179-1204.

Claret, C., P. Marmonier, M. J. Dole-Olivier, M. C. Des Chatelliers, A. J. Boulton & E. Castella, 1999b. A functional classification of interstitial invertebrates: supplementing measures of biodiversity using species traits and habitat affinities. Archiv Fur Hydrobiologie 145: 385-403.

Clements, A. R., P. J. Suter, M. Fussell & E. Silvester, 2016. Macroinvertebrate communities in spring-fed alpine source pools. Hydrobiologia 777: 119-138.

Cook, B. D., K. M. Abrams, J. Marshall, C. N. Perna, S. Choy, M. T. Guzik & S. J. B. Cooper, 2012. Species diversity and genetic differentiation of stygofauna (Syncarida: Bathynellacea) across an alluvial aquifer in north-eastern Australia. Australian Journal of Zoology 60: 152-158.

Cottarelli, V., M. C. Bruno & R. Berera, 2008. Two new species of Parastenocaris (Copepoda, Harpacticoida, Parastenocarididae) from groundwater of Sardinia and Sicily. Crustaceana 81: 537-555.

Cottarelli, V., M. C. Bruno, M. T. Spena & R. Grasso, 2012. Studies on Subterranean Copepods from Italy, with Descriptions of Two New Epikarstic Species from a Cave in Sicily. Zoological Studies 51: 556-582.

Culver, D. C., J. R. Holsinger & D. J. Feller, 2012. The Fauna of Seepage Springs and Other Shallow Subterranean Habitats in the Mid-Atlantic Piedmont and Coastal Plain. Northeastern Naturalist 19: 1-42.

Culver, D. C. & T. Pipan, 2011. Redefining the extent of the aquatic subterranean biotope-shallow subterranean habitats. Ecohydrology 4: 721-730.

Curcic, B. P. M., 2008. Ayyalonia dimentmani n. g., n. sp (Ayyaloniini n. trib., Chthoniidae, Pseudoscorpiones) from a cave in Israel. Archives of Biological Sciences 60: 331-339.

Danielopol, D. L., 1989. Groundwater Fauna Associated with Riverine Aquifers. Journal of the North American Benthological Society 8: 18-35.

Danielopol, D. L. & P. Pospisil, 2001. Hidden biodiversity in the groundwater of the Danube Flood Plain National Park (Austria). Biodiversity and Conservation 10: 1711-1721.

Danielopol, D. L. & P. Pospisil, 2002. Taxonomic diversity of Crustacea Cyclopoida in the Austrian "Danube Floodplain" National Park. Vie Et Milieu-Life and Environment 52: 67-75.

Danielopol, D. L., R. Rouch & A. Baltanas, 2002. Taxonomic diversity of groundwater harpacticoida (Copepoda, Crustacea) in southern France - A contribution to characterise hotspot diversity sites. Vie Et Milieu-Life and Environment 52: 1-15.

Danks, H. V. & D. D. Williams, 1991. Arthropods of Springs, with Particular Reference to Canada - Synthesis and Needs for Research. Memoirs of the Entomological Society of Canada: 203-217.

Darocha, C. E. F. & C. C. Hakenkamp, 1993. New Species of Halicyclops (Copepoda, Cyclopidae) from the United-States-of-America. Hydrobiologia 259: 145-156.

Di Lorenzo, T., M. Cifoni, P. Lombardo, B. Fiasca & D. M. P. Galassi, 2015. Ammonium threshold values for groundwater quality in the EU may not protect groundwater fauna: evidence from an alluvial aquifer in Italy. Hydrobiologia 743: 139-150.

Di Lorenzo, T., D. Cipriani, B. Fiasca, S. Rusi & D. M. P. Galassi, 2018. Groundwater drift monitoring as a tool to assess the spatial distribution of groundwater species into karst aquifers. Hydrobiologia 813: 137-156.

Di Lorenzo, T., W. D. Di Marzio, B. Fiasca, D. M. P. Galassi, K. Korbel, S. Iepure, J. L. Pereira, A. S. P. S. Reboleira, S. I. Schmidt & G. C. Hose, 2019. Recommendations for ecotoxicity testing with stygobiotic species in the framework of groundwater environmental risk assessment. Science of the Total Environment 681: 292-304.

Di Lorenzo, T. & D. M. P. Galassi, 2013. Agricultural impact on Mediterranean alluvial aquifers: do groundwater communities respond? Fundamental and Applied Limnology 182: 271-282.

Di Lorenzo, T. & D. M. P. Galassi, 2017. Effect of Temperature Rising on the Stygobitic Crustacean Species Diacyclops belgicus: Does Global Warming Affect Groundwater Populations? Water 9.

Dole-Olivier, M. J., 1998. Surface water-groundwater exchanges in three dimensions on a backwater of the Rhone River. Freshwater Biology 40: 93-109.

Dole-Olivier, M. J., F. Castellarini, N. Coineau, D. M. P. Galassi, P. Martin, N. Mori, A. Valdecasas & J. Gibert, 2009a. Towards an optimal sampling strategy to assess groundwater biodiversity: comparison across six European regions. Freshwater Biology 54: 777-796.

Dole-Olivier, M. J., D. M. P. Galassi, F. Fiers, F. Malard, P. Martin, D. Martin & P. Marmonier, 2015. Biodiversity in mountain groundwater: the Mercantour National Park (France) as a European hotspot. Zoosystema 37: 529-550.

Dole-Olivier, M. J., C. Maazouzi, B. Cellot, F. Fiers, D. M. P. Galassi, C. Claret, D. Martin, S. Merigoux & P. Marmonier, 2014. Assessing invertebrate assemblages in the subsurface zone of stream sediments ( 0-15 cm deep) using a hyporheic sampler. Water Resources Research 50: 453-465.

Dole-Olivier, M. J., F. Malard, D. Ferreira & J. Gibert, 2005. Groundwater biodiversity. Houille Blanche-Revue Internationale De L Eau: 39-44.

Dole-Olivier, M. J., F. Malard, D. Martin, T. Lefebure & J. Gibert, 2009b. Relationships between environmental variables and groundwater biodiversity at the regional scale. Freshwater Biology 54: 797-813.

Doleolivier, M. J. & P. Marmonier, 1992a. Effects of Spates on the Vertical-Distribution of the Interstitial Community. Hydrobiologia 230: 49-61.

Doleolivier, M. J. & P. Marmonier, 1992b. Patch Distribution of Interstitial Communities - Prevailing Factors. Freshwater Biology 27: 177-191.

Dumas, P., 2002. Stability of interstitial crustacean communities in an isolated alluvial aquifer. Hydrobiologia 468: 63-76.

Dumas, P., 2004. Irrigation as a disturbance for interstitial crustacean communities in a French Pyrenean alluvial aquifer. Annales De Limnologie-International Journal of Limnology 40: 139-147.

Dumas, P., C. Bou & J. Gibert, 2001. Groundwater macrocrustaceans as natural indicators of the Ariege alluvial aquifer. International Review of Hydrobiology 86: 619-633.

Dumas, P. & G. Fontanini, 2001. Sampling fauna in aquifers: a comparison of net-sampling and pumping. Archiv Fur Hydrobiologie 150: 661-676.

Dumnicka, E., J. Galas & M. Krodkiewska, 2017. Patterns of Benthic Fauna Distribution in Wells: The Role of Anthropogenic Impact and Geology. Vadose Zone Journal 16.

Dumont, H. J. & S. Negrea, 1996. A conspectus of the Cladocera of the subterranean waters of the world. Hydrobiologia 325: 1-30.

Durkota, J. M., P. J. Wood, T. Johns, J. R. Thompson & R. J. Flower, 2019. Distribution of macroinvertebrate communities across surface and groundwater habitats in response to hydrological variability. Fundamental and Applied Limnology 193: 79-92.

Eberhard, S. M., S. A. Halse, M. R. Williams, M. D. Scanlon, J. Cocking & H. J. Barron, 2009. Exploring the relationship between sampling efficiency and short-range endemism for groundwater fauna in the Pilbara region, Western Australia. Freshwater Biology 54: 885-901.

Eisendle-Flockner, U. & S. Hilberg, 2015. Hard rock aquifers and free-living nematodes - an interdisciplinary approach based on two widely neglected components in groundwater research. Ecohydrology 8: 368-377.

El Adnani, M., A. A. Boughrous, M. Y. Khebiza, A. El Gharmali, M. L. Sbai, A. S. Errouane, L. L. Idrissi & A. Nejmeddine, 2007. Impact of mining wastes on the physicochemical and biological characteristics of groundwater in a mining area in Marrakech (Morocco). Environmental Technology 28: 71-82.

Engel, A. S., 2007. Observations on the biodiversity of sulfidic karst habitats. Journal of Cave and Karst Studies 69: 187-206.

Ercoli, F., F. Lefebvre, M. Delangle, N. Gode, M. Caillon, R. Raimond & C. Souty-Grosset, 2019. Differing trophic niches of three French stygobionts and their implications for conservation of endemic stygofauna. Aquatic Conservation-Marine and Freshwater Ecosystems.

Erseus, C., 1992. Groundwater and Marine Intertidal Tubificidae (Oligochaeta) from the Canary-Islands and Cabo Verde Islands, with Descriptions of 2 New Species. Bijdragen Tot De Dierkunde 62: 63-70.

Fenolio, D. B., M. L. Niemiller, A. G. Gluesenkamp, A. M. McKee & S. J. Taylor, 2017. New Distributional Records of the Stygobitic Crayfish Cambarus cryptodytes (Decapoda: Cambaridae) in the Floridan Aquifer System of Southwestern Georgia. Southeastern Naturalist 16: 163-181.

Fenwick, G. D., 2001. The freshwater Amphipoda (Crustacea) of New Zealand: a review. Journal of the Royal Society of New Zealand 31: 341-363.

Ferreira, D., F. Malard, M. J. Dole-Olivier & J. Gibert, 2007. Obligate groundwater fauna of France: diversity patterns and conservation implications. Biodiversity and Conservation 16: 567-596.

Finston, T. L., C. J. Francis & M. S. Johnson, 2009. Biogeography of the stygobitic isopod Pygolabis (Malacostraca: Tainisopidae) in the Pilbara, Western Australia: Evidence for multiple colonisations of the groundwater. Molecular Phylogenetics and Evolution 52: 448-460.

Finston, T. L., S. S. Lukehurst & G. L. Fitzpatrick, 2010. Characterisation of microsatellite loci in the groundwater amphipod Chydaekata sp. (Malacostraca: Paramelitidae). Conservation Genetics Resources 2: 237-239.

Fiser, C., R. Alther, V. Zaksek, S. Borko, A. Fuchs & F. Altermatt, 2018a. Translating Niphargus barcodes from Switzerland into taxonomy with a description of two new species (Amphipoda, Niphargidae). Zookeys: 113-141.

Fiser, C., M. Zagmajster & M. Dethier, 2018b. Overview of Niphargidae (Crustacea: Amphipoda) in Belgium: distribution, taxonomic notes and conservation issues. Zootaxa 4387: 47-74.

Foulquier, A., F. Malard, T. Lefebure, J. Gibert & C. J. Douady, 2008. The imprint of Quaternary glaciers on the present-day distribution of the obligate groundwater amphipod Niphargus virei (Niphargidae). Journal of Biogeography 35: 552-564.

Foulquier, A., L. Simon, F. Gilbert, F. Fourel, F. Malard & F. Mermillod-Blondin, 2010. Relative influences of DOC flux and subterranean fauna on microbial abundance and activity in aquifer sediments: new insights from 13C-tracer experiments. Freshwater Biology 55: 1560-1576.

Fowler, R. T. & M. R. Scarsbrook, 2002. Influence of hydrologic exchange patterns on water chemistry and hyporheic invertebrate communities in three gravel-bed rivers. New Zealand Journal of Marine and Freshwater Research 36: 471-482.

Fraser, B. G. & D. D. Williams, 1998. Seasonal boundary dynamics of a groundwater/surface-water ecotone. Ecology 79: 2019-2031.

Gabrovsek, F., M. Knez, J. Kogovsek, A. Mihevc, J. Mulec, M. Perne, M. Petric, T. Pipan, M. Prelovsek, T. Slabe, S. Sebela & N. Ravbar, 2011. Development challenges in karst regions: sustainable land use planning in the karst of Slovenia. Carbonates and Evaporites 26: 365-380.

Galassi, D. M. P., 2001. Groundwater copepods: diversity patterns over ecological and evolutionary scales. Hydrobiologia 453: 227-253.

Galassi, D. M. P., R. Huys & J. W. Reid, 2009. Diversity, ecology and evolution of groundwater copepods. Freshwater Biology 54: 691-708.

Galassi, D. M. P., P. Lombardo, B. Fiasca, A. Di Cioccio, T. Di Lorenzo, M. Petitta & P. Di Carlo, 2014. Earthquakes trigger the loss of groundwater biodiversity. Scientific Reports 4.

Galassi, D. M. P., F. Stoch & A. Brancelj, 2013. Dissecting copepod diversity at different spatial scales in southern European groundwater. Journal of Natural History 47: 821-840.

Garman, K. M., H. Rubelmann, D. J. Karlen, T. H. Wu & J. R. Garey, 2011. Comparison of an inactive submarine spring with an active nearshore anchialine spring in Florida. Hydrobiologia 677: 65-87.

Gerecke, R., P. Martin & T. Gledhill, 2018. Water mites (Acari: Parasitengona: Hydrachnidia) as inhabitants of groundwater-influenced habitats - considerations following an update of Limnofauna Europaea. Limnologica 69: 81-93.

Ghlala, A., D. Della Valle & G. Messana, 2009. First record of the genus Typhlocirolana Racovitza, 1905 (Isopoda: Cirolanidae) from Tunisia and description of a new species from the National park of Ichkeul. Zootaxa: 57-64.

Giani, N., B. Sambugar, P. Rodriguez & E. Martinez-Ansemil, 2001. Oligochaetes in southern European groundwater: new records and an overview. Hydrobiologia 463: 65-74.

Gibert, J., D. C. Culver, M. J. Dole-Olivier, F. Malard, M. C. Christman & L. Deharveng, 2009. Assessing and conserving groundwater biodiversity: synthesis and perspectives. Freshwater Biology 54: 930-941.

Gibert, J., P. Marmonier, V. Vanek & S. Plenet, 1995. Hydrological exchange and sediment characteristics in a riverbank: Relationship between heavy metals and invertebrate community structure. Canadian Journal of Fisheries and Aquatic Sciences 52: 2084-2097.

Gibson, L., W. F. Humphreys, M. Harveyc, B. Hyder & A. Winzer, 2019. Shedding light on the hidden world of subterranean fauna: A transdisciplinary research approach. Science of the Total Environment 684: 381-389.

Glasstetter, M., 2012. Earthworm diversity in urban habitats of Basel (Northwestern Switzerland) (Oligochaeta: Lumbricidae). Zoology in the Middle East: 95-102.

Goricki, S., D. Stankovic, A. Snoj, M. Kuntner, W. R. Jeffery, P. Trontelj, M. Pavicevic, Z. Grizelj, M. Naparus-Aljancic & G. Aljancic, 2017. Environmental DNA in subterranean biology: range extension and taxonomic implications for Proteus. Scientific Reports 7.

Gossel, W., R. Lahne & C. Neumann, 2007. Ground water animals - world wide one time survey for groundwater fauna successfully closed. Grundwasser 12: 254-256.

Gottstein, S., M. Ivkovic, I. Ternjej, B. Jalzic & M. Kerovec, 2007. Environmental features and crustacean community of anchihaline hypogean waters on the Kornati islands, Croatia. Marine Ecology-an Evolutionary Perspective 28: 24-30.

Graillot, D., F. Paran, G. Bornette, P. Marmonier, C. Piscart & L. Cadilhac, 2014. Coupling groundwater modeling and biological indicators for identifying river/aquifer exchanges. Springerplus 3.

Graymore, M., F. Stagnitti & G. Allinson, 2001. Impacts of atrazine in aquatic ecosystems. Environment International 26: 483-495.

Griebler, C., H. Brielmann, C. M. Haberer, S. Kaschuba, C. Kellermann, C. Stumpp, F. Hegler, D. Kuntz, S. Walker-Hertkorn & T. Lueders, 2016. Potential impacts of geothermal energy use and storage of heat on groundwater quality, biodiversity, and ecosystem processes. Environmental Earth Sciences 75.

Griebler, C., K. Hug, L. Fillinger, A. Meyer & M. Avramov, 2018. The B-A-E index - a microbiological-ecological concept for the assessment and monitoring of groundwater. Hydrologie Und Wasserbewirtschaftung 62: 378-386.

Griebler, C., F. Malard & T. Lefebure, 2014. Current developments in groundwater ecology - from biodiversity to ecosystem function and services. Current Opinion in Biotechnology 27: 159-167.

Griebler, C., H. Stein, C. Kellermann, S. Berkhoff, H. Brielmann, S. Schmidt, D. Selesi, C. Steube, A. Fuchs & H. J. Hahn, 2010. Ecological assessment of groundwater ecosystems - Vision or illusion? Ecological Engineering 36: 1174-1190.

Gunn, J., P. Hardwick & P. J. Wood, 2000. The invertebrate community of the Peak-Speedwell cave system, Derbyshire, England pressures and considerations for conservation management. Aquatic Conservation-Marine and Freshwater Ecosystems 10: 353-369.

Gutjahr, S., J. Bork & H. J. Hahn, 2013a. Groundwater fauna as an indicator for complex hydrogeological conditions in the Western Kaiserstuhl. Grundwasser 18: 173-184.

Gutjahr, S., J. Bork, S. I. Schmidt & H. J. Hahn, 2013b. Efficiency of sampling invertebrates in groundwater habitats. Limnologica 43: 43-48.

Guy-Haim, T., N. Simon-Blecher, A. Frumkin, I. Naaman & Y. Achituv, 2018. Multiple transgressions and slow evolution shape the phylogeographic pattern of the blind cave-dwelling shrimp Typhlocaris. Peerj 6.

Guzik, M. T., A. D. Austin, S. J. B. Cooper, M. S. Harvey, W. F. Humphreys, T. Bradford, S. M. Eberhard, R. A. King, R. Leys, K. A. Muirhead & M. Tomlinson, 2010. Is the Australian subterranean fauna uniquely diverse? Invertebrate Systematics 24: 407-418.

Guzik, M. T., S. J. B. Cooper, W. F. Humphreys, S. Ong, T. Kawakami & A. D. Austin, 2011. Evidence for population fragmentation within a subterranean aquatic habitat in the Western Australian desert. Heredity 107: 215-230.

Guzik, M. T., D. N. Stringer, N. P. Murphy, S. J. B. Cooper, S. Taiti, R. A. King, W. F. Humphreys & A. D. Austin, 2019. Molecular phylogenetic analysis of Australian arid-zone oniscidean isopods (Crustacea:Haloniscus) reveals strong regional endemicity and new putative species. Invertebrate Systematics 33: 556-574.

Hahn, H. J., 2003. Are traps suitable for representative sampling of aquatic meiofauna in both the hyporheic zone and in groundwater? Limnologica 33: 138-146.

Hahn, H. J., 2006. The GW-Fauna-Index: A first approach to a quantitative ecological assessment of groundwater habitats. Limnologica 36: 119-137.

Hahn, H. J. & A. Fuchs, 2009. Distribution patterns of groundwater communities across aquifer types in south-western Germany. Freshwater Biology 54: 848-860.

Hahn, H. J. & S. Gutjahr, 2014. Bio Indication in Groundwater works - Reply to Comment by T. Scheytt on the Report "Groundwater Fauna as an Indicator for complex hydrogeological Conditions at the western Kaiserstuhl". Grundwasser 19: 215-218.

Hahn, H. J. & D. Matzke, 2005. A comparison of stygofauna communities inside and outside groundwater bores. Limnologica 35: 31-44.

Hahn, H. R., 2005. Unbaited phreatic traps: A new method of sampling stygofauna. Limnologica 35: 248-261.

Hallam, F., M. Yacoubi-Khebiza, K. Oufdou & M. Boulanouar, 2008. Groundwater Quality in an Arid Area of Morocco: Impact of Pollution on the Biodiversity and Relationships between Crustaceans and Bacteria of Health Interest. Environmental Technology 29: 1179-1189.

Hancock, P. J. & A. J. Boulton, 2008. Stygofauna biodiversity and endemism in four alluvial aquifers in eastern Australia. Invertebrate Systematics 22: 117-126.

Hancock, P. J. & A. J. Boulton, 2009. Sampling groundwater fauna: efficiency of rapid assessment methods tested in bores in eastern Australia. Freshwater Biology 54: 902-917.

Hancock, P. J., A. J. Boulton & W. F. Humphreys, 2005. Aquifers and hyporheic zones: Towards an ecological understanding of groundwater. Hydrogeology Journal 13: 98-111.

Harvey, M. S., M. G. Rix, V. W. Framenau, Z. R. Hamilton, M. S. Johnson, R. J. Teale, G. Humphreys & W. F. Humphreys, 2011. Protecting the innocent: studying short-range endemic taxa enhances conservation outcomes. Invertebrate Systematics 25: 1-10.

Hilberg, S. & U. Eisendle-Flockner, 2016. About faunal life in Austrian aquifers - historical background and current developments. Austrian Journal of Earth Sciences 109.

Hof, C., M. Brandle & R. Brandl, 2008. Latitudinal variation of diversity in European freshwater animals is not concordant across habitat types. Global Ecology and Biogeography 17: 539-546.

Holsinger, J. R., 1993. Biodiversity of Subterranean Amphipod Crustaceans - Global Patterns and Zoogeographic Implications. Journal of Natural History 27: 821-835.

Hose, G. C., 2005. Assessing the need for groundwater quality guidelines for pesticides using the species sensitivity distribution approach. Human and Ecological Risk Assessment 11: 951-966.

Hose, G. C., K. A. Fryirs, J. Bailey, N. Ashby, T. White & C. Stumpp, 2017. Different depths, different fauna: habitat influences on the distribution of groundwater invertebrates. Hydrobiologia 797: 145-157.

Hose, G. C., K. Symington, M. J. Lott & M. J. Lategan, 2016. The toxicity of arsenic(III), chromium(VI) and zinc to groundwater copepods. Environmental Science and Pollution Research 23: 18704-18713.

Huebner, C. B. & M. R. Vinson, 2004. Groundwater invertebrate fauna of the Bear River Range, Utah. Western North American Naturalist 64: 131-134.

Humphreys, W. F., 2001. Milyeringa veritas (Eleotridae), a remarkably versatile cave fish from the arid tropics of northwestern Australia. Environmental Biology of Fishes 62: 297-313.

Humphreys, W. F., 2006. Aquifers: the ultimate groundwater-dependent ecosystems. Australian Journal of Botany 54: 115-132.

Humphreys, W. F., 2008. Rising from Down Under: developments in subterranean biodiversity in Australia from a groundwater fauna perspective. Invertebrate Systematics 22: 85-101.

Humphreys, W. F., 2014. Subterranean fauna of Christmas Island: habitats and salient features. Raffles Bulletin of Zoology: 29-44.

Hutchins, B. & W. Orndorff, 2009. Effectiveness and Adequacy of Well Sampling Using Baited Traps for Monitoring the Distribution and Abundance of an Aquatic Subterranean Isopod. Journal of Cave and Karst Studies 71: 193-203.

Hutchins, B. T., 2018. The conservation status of Texas groundwater invertebrates. Biodiversity and Conservation 27: 475-501.

Hyde, J., S. J. B. Cooper, W. F. Humphreys, A. D. Austin & P. Munguia, 2018. Diversity patterns of subterranean invertebrate fauna in calcretes of the Yilgarn Region, Western Australia. Marine and Freshwater Research 69: 114-121.

Iepure, S., A. Feurdean, C. Badaluta, V. Nagavciuc & A. Persoiu, 2016. Pattern of richness and distribution of groundwater Copepoda (Cyclopoida: Harpacticoida) and Ostracoda in Romania: an evolutionary perspective. Biological Journal of the Linnean Society 119: 593-608.

Iepure, S., R. Rasines-Ladero, R. Meffe, F. Carreno, D. Mostaza, A. Sundberg, T. Di Lorenzo & J. L. Barroso, 2017. Exploring the distribution of groundwater Crustacea (Copepoda and Ostracoda) to disentangle aquifer type featuresA case study in the upper Tajo basin (Central Spain). Ecohydrology 10.

Issartel, J., Y. Voituron, O. Guillaume, J. Clobert & F. Hervant, 2010. Selection of physiological and metabolic adaptations to food deprivation in the Pyrenean newt Calotriton asper during cave colonisation. Comparative Biochemistry and Physiology a-Molecular & Integrative Physiology 155: 77-83.

Jasinska, E. J., B. Knott & A. J. McComb, 1996. Root mats in ground water: A fauna-rich cave habitat. Journal of the North American Benthological Society 15: 508-519.

Jaume, D., 2008. Global diversity of spelaeogriphaceans & thermosbaenaceans (Crustacea; Spelaeogriphacea & Thermosbaenacea) in freshwater. Hydrobiologia 595: 219-224.

Javidkar, M., S. J. B. Cooper, W. F. Humphreys, R. A. King, S. Judd & A. D. Austin, 2018. Biogeographic history of subterranean isopods from groundwater calcrete islands in Western Australia. Zoologica Scripta 47: 206-220.

Javidkar, M., S. J. B. Cooper, R. A. King, W. F. Humphreys, T. Bertozzi, M. I. Stevens & A. D. Austin, 2016. Molecular systematics and biodiversity of oniscidean isopods in the groundwater calcretes of central Western Australia. Molecular Phylogenetics and Evolution 104: 83-98.

Javidkar, M., R. A. King, S. J. B. Cooper, W. F. Humphreys & A. D. Austin, 2017. Taxonomy of Paraplatyarthrus Javidkar and King (Isopoda: Oniscidea: Paraplatyarthridae) with description of five new species from Western Australia, and comments on Australian Trichorhina Budde-Lunde, 1908 (Platyarthridae). Zootaxa 4243: 401-431.

Johns, T., J. I. Jones, L. Knight, L. Maurice, P. Wood & A. Robertson, 2015. Regional-scale drivers of groundwater faunal distributions. Freshwater Science 34: 316-328.

Juget, J. & M. YacoubiKhebiza, 1997. Research on the ecology of Astacopsidrilus naceri Giani & Martin, 1995 (Phreodrilidae, Oligochaeta) from the groundwaters of Morocco. Annales De Limnologie-International Journal of Limnology 33: 149-161.

Karanovic, I. & K. McKay, 2010. Two new species of Leicacandona Karanovic (Ostracoda, Candoninae) from the Great Sandy Desert, Australia. Journal of Natural History 44: 2715-2736.

Karanovic, T. & S. J. B. Cooper, 2011. Molecular and morphological evidence for short range endemism in the Kinnecaris solitaria complex (Copepoda: Parastenocarididae), with descriptions of seven new species. Zootaxa: 1-64.

Karanovic, T. & S. J. B. Cooper, 2012. Explosive radiation of the genus Schizopera on a small subterranean island in Western Australia (Copepoda : Harpacticoida): unravelling the cases of cryptic speciation, size differentiation and multiple invasions. Invertebrate Systematics 26: 115-192.

Karanovic, T., S. Eberhard, S. J. B. Cooper & M. T. Guzik, 2015. Morphological and molecular study of the genus Nitokra (Crustacea, Copepoda, Harpacticoida) in a small palaeochannel in Western Australia. Organisms Diversity & Evolution 15: 65-99.

Karanovic, T., S. M. Eberhard & A. Murdoch, 2011. A Cladistic Analysis and Taxonomic Revision of Australian Metacyclops and Goniocyclops, with Description of Four New Species and Three New Genera (Copepoda, Cyclopoida). Crustaceana 84: 1-67.

Karanovic, T., S. M. Eberhard, G. Perina & S. Callan, 2013. Two new subterranean ameirids (Crustacea : Copepoda : Harpacticoida) expose weaknesses in the conservation of short-range endemics threatened by mining developments in Western Australia. Invertebrate Systematics 27: 540-566.

Karanovic, T. & M. Krajicek, 2012. First Molecular Data on the Western Australian Diacyclops (Copepoda, Cyclopoida) Confirm Morpho-Species but Question Size Differentiation and Monophyly of the Alticola-Group. Crustaceana 85: 1549-1569.

Kaser, D., 2010. A new habitat of subsurface waters: the hyporheic biotope. Fundamental and Applied Limnology 176: 291-302.

Kayo, R. T., P. Marmonier, S. H. Z. Togouet, M. Nola & C. Piscart, 2012. An Annotated Checklist of Freshwater Stygobiotic Crustaceans of Africa and Madagascar. Crustaceana 85: 1613-1631.

Khebiza, M. Y., A. A. Boughrous, C. Gabbanini, M. Messouli & G. Messana, 2006. Impact of waste discharges on the water quality and interstitial community structure of two Mediterranean rivers. Italian Journal of Zoology 73: 153-166.

Kibichii, S., J. R. Baars & M. Kelly-Quinn, 2009. Optimising sample volume and replicates using the Bou-Rouch method for the rapid assessment of hyporheic fauna. Marine and Freshwater Research 60: 83-96.

Kibichii, S., H. B. Feeley, J. R. Baars & M. Kelly-Quinn, 2015. The influence of water quality on hyporheic invertebrate communities in agricultural catchments. Marine and Freshwater Research 66: 805-814.

King, R. A., T. Bradford, A. D. Austin, W. F. Humphreys & S. J. B. Cooper, 2012. Divergent Molecular Lineages and Not-So-Cryptic Species: The First Descriptions of Stygobitic Chiltoniid Amphipods (Talitroidea: Chiltoniidae) from Western Australia. Journal of Crustacean Biology 32: 465-488.

Knight, L. R. F. D. & M. R. Penk, 2010. Groundwater Crustacea of Ireland: A Survey of the Stygobitic Malacostraca in Caves and Springs. Biology and Environment-Proceedings of the Royal Irish Academy 110b: 211-235.

Konec, M., T. Delic & P. Trontelj, 2016. DNA barcoding sheds light on hidden subterranean boundary between Adriatic and Danubian drainage basins. Ecohydrology 9: 1304-1312.

Korbel, K., A. Chariton, S. Stephenson, P. Greenfield & G. C. Hose, 2017. Wells provide a distorted view of life in the aquifer: implications for sampling, monitoring and assessment of groundwater ecosystems. Scientific Reports 7.

Korbel, K. L., S. Stephenson & G. C. Hose, 2019. Sediment size influences habitat selection and use by groundwater macrofauna and meiofauna. Aquatic Sciences 81.

Kulkotyluoglu, O., M. Yavuzatmaca, D. Akdemir, B. F. Schwartz & B. T. Hutchins, 2017. Ufocandona hannaleeae gen. et sp nov (Crustacea, Ostracoda) from an artesian well in Texas, USA. European Journal of Taxonomy 372.

Lafont, M., J. C. Camus & A. Rosso, 1996. Superficial and hyporheic oligochaete communities as indicators of pollution and water exchange in the River Moselle, France. Hydrobiologia 334: 147-155.

Lafont, M. & A. Vivier, 2006. Oligochaete assemblages in the hyporheic zone and coarse surface sediments: Their importance for understanding of ecological functioning of watercourses. Hydrobiologia 564: 171-181.

Lafont, M., A. Vivier, S. Nogueira, P. Namour & P. Breil, 2006. Surface and hyporheic oligochaete assemblages in a French suburban stream. Hydrobiologia 564: 183-193.

Lagnika, M., M. Ibikounle, C. Boutin & N. G. Sakiti, 2016. Groundwater biodiversity and water quality of wells in the Southern region of Benin. Comptes Rendus Chimie 19: 798-806.

Leijs, R., A. Bloechl & S. Koenemann, 2011. Bogidiella Veneris, a New Species of Subterranean Amphipoda (Bogidiellidae) from Australia, with Remarks on the Systematics and Biogeography. Journal of Crustacean Biology 31: 566-575.

Lewis, J. J., G. O. Graening, D. B. Fenolio & E. A. Bergey, 2006. Caecidotea mackini, new species, with a synopsis of the subterranean asellids of Oklahoma (Crustacea : Isopoda : Asellidae). Proceedings of the Biological Society of Washington 119: 563-575.

Lewis, J. J. & J. W. Reid, 2007. Patterns and processes of groundwater invasion by copepods in the interior low plateaus of the United States. Acta Carsologica 36: 279-289.

Little, J., D. J. Schmidt, B. D. Cook, T. J. Page & J. M. Hughes, 2016. Diversity and phylogeny of south-east Queensland Bathynellacea. Australian Journal of Zoology 64: 36-47.

Liu, W., C. Y. Zhou, J. E. Burnet & A. Brancelj, 2017. The effect of hydrological and hydrochemical parameters on the microdistribution of aquatic fauna in drip water in the Velika Pasica Cave, Central Slovenia. Ecohydrology 10.

Lupkes, G., 1974. 3 New Stygobiont Peritricha from Interstitial Groundwater Contribution to Knowledge About Stygobiont Peritrich Fauna of Germany. Archiv Fur Hydrobiologie 73: 394-402.

MacKay, S. E. & D. D. Williams, 2011. Invertebrate colonization of the surface and deep groundwaters of a small oceanic island (Barbados, West Indies). Tropical Zoology 24: 1-47.

Mahi, A., T. Di Lorenzo, B. Haicha, N. Belaidi & A. Taleb, 2019. Environmental factors determining regional biodiversity patterns of groundwater fauna in semi-arid aquifers of northwest Algeria. Limnology 20: 309-320.

Maitland, P. S., 1999. New horizons - new species? The invertebrate fauna of unexplored aquatic habitats in Scotland. Aquatic Conservation-Marine and Freshwater Ecosystems 9: 529-534.

Malard, F., C. Boutin, A. I. Camacho, D. Ferreira, G. Michel, B. Sket & F. Stoch, 2009. Diversity patterns of stygobiotic crustaceans across multiple spatial scales in Europe. Freshwater Biology 54: 756-776.

Malard, F., C. Capderrey, B. Churcheward, D. Eme, B. Kaufmann, L. Konecny-Dupre, J. P. Lena, F. Liebault & C. J. Douady, 2017. Geomorphic influence on intraspecific genetic differentiation and diversity along hyporheic corridors. Freshwater Biology 62: 1955-1970.

Malard, F., D. Ferreira, S. Doledec & J. V. Ward, 2003a. Influence of groundwater upwelling on the distribution of the hyporheos in a headwater river flood plain. Archiv Fur Hydrobiologie 157: 89-116.

Malard, F., D. Galassi, M. Lafont, S. Doledec & J. V. Ward, 2003b. Longitudinal patterns of invertebrates in the hyporheic zone of a glacial river. Freshwater Biology 48: 1709-1725.

Malard, F., J. Gibert, R. Laurent & J. L. Reygrobellet, 1994. A New Method for Sampling the Fauna of Deep Karstic Aquifers. Comptes Rendus De L Academie Des Sciences Serie Iii-Sciences De La Vie-Life Sciences 317: 955-966.

Malard, F., J. Mathieu, J. L. Reygrobellet & M. Lafont, 1996. Biomotoring groundwater contamination: Application to a karst area in Southern France. Aquatic Sciences 58: 158-187.

Malard, F., J. L. Reygrobellet, R. Laurent & J. Mathieu, 1997. Developments in sampling the fauna of deep water-table aquifers. Archiv Fur Hydrobiologie 138: 401-432.

Mammola, S. & B. Leroy, 2018. Applying species distribution models to caves and other subterranean habitats. Ecography 41: 1194-1208.

Manenti, R., 2014. Role of Cave Features for Aquatic Troglobiont Fauna Occurrence: Effects on "Accidentals" and Troglomorphic Organisms Distribution. Acta Zoologica Academiae Scientiarum Hungaricae 60: 257-270.

Marmonier, P., C. Claret & M. J. Dole-Olivier, 2000. Interstitial fauna in newly-created floodplain canals of a large regulated river. Regulated Rivers-Research & Management 16: 23-36.

Marmonier, P., C. Maazouzi, A. Foulquier, S. Navel, C. Francois, F. Hervant, F. Mermillod-Blondin, A. Vieney, S. Barraud, A. Togola & C. Piscart, 2013. The use of crustaceans as sentinel organisms to evaluate groundwater ecological quality. Ecological Engineering 57: 118-132.

Marmonier, P., M. J. Olivier, M. C. D. des Chatelliers, F. Paran, D. Graillot, T. Winiarski, L. Konecny-Dupre, S. Navel & L. Cadilhac, 2019. Does spatial heterogeneity of hyporheic fauna vary similarly with natural and artificial changes in braided river width? Science of the Total Environment 689: 57-69.

Marmonier, P., P. Vervier, J. Gibert & M. J. Doleolivier, 1993. Biodiversity in-Ground Waters. Trends in Ecology & Evolution 8: 392-395.

Martin, P., C. De Broyer, F. Fiers, G. Michel, R. Sablon & K. Wouters, 2009. Biodiversity of Belgian groundwater fauna in relation to environmental conditions. Freshwater Biology 54: 814-829.

Martin, P., R. M. Schmelz & M. J. Dole-Olivier, 2015. Groundwater oligochaetes (Annelida, Clitellata) from the Mercantour National Park (France), with the descriptions of one new genus and two new stygobiont species. Zoosystema 37: 551-569.

Martinez-Ansemil, E., F. Giacomazzi & B. Sambugar, 2016. Groundwater oligochaetes (Annelida: Clitellata) of the Dinaric region (South-East Europe). Biologia 71: 24-30.

Martinez-Ansemil, E., N. Giani & B. Sambugar, 2002. Oligochaetes from underground waters of Oman with descriptions of two new species of Phreodrilidae (Oligochaeta): Antarctodrilus arabicus n sp and Phreodrilus stocki n sp. Contributions to Zoology 71: 147-158.

Masciopinto, C., F. Semeraro, R. La Mantia, S. Inguscio & E. Rossi, 2006. Stygofauna abundance and distribution in the fissures and caves of the Nardo (southern Italy) fractured aquifer subject to reclaimed water injections. Geomicrobiology Journal 23: 267-278.

Mauclaire, L. & J. Gibert, 2001. Environmental determinants of bacterial activity and faunal assemblages in alluvial riverbank aquifers. Archiv Fur Hydrobiologie 152: 469-487.

Mauclaire, L., P. Marmonier & J. Gibert, 1998. Sampling water and sediment in interstitial habitats: a comparison of coring and pumping techniques. Archiv Fur Hydrobiologie 142: 111-123.

Maurice, L., A. R. Robertson, D. White, L. Knight, T. Johns, F. Edwards, M. Arietti, J. P. R. Sorensen, D. Weitowitz, B. P. Marchant & J. P. Bloomfield, 2016. The invertebrate ecology of the Chalk aquifer in England (UK). Hydrogeology Journal 24: 459-474.

Mcinerney, C. E., L. Maurice, A. L. Robertson, L. R. F. D. Knight, J. Arnscheidt, C. Venditti, J. S. G. Dooley, T. Mathers, S. Matthijs, K. Eriksson, G. S. Proudlove & B. Hanfling, 2014. The ancient Britons: groundwater fauna survived extreme climate change over tens of millions of years across NW Europe. Molecular Ecology 23: 1153-1166.

Meland, K., J. Mees, M. Porter & K. J. Wittmann, 2015. Taxonomic Review of the Orders Mysida and Stygiomysida (Crustacea, Peracarida). Plos One 10.

Meleg, I. N., K. P. Battes, F. Fiers & O. T. Moldovan, 2015. Contrasting copepod community dynamics related to sampling strategies in the unsaturated zone of a karst aquifer. Aquatic Ecology 49: 549-560.

Meleg, I. N., F. Fiers, M. Robu & O. T. Moldovan, 2012. Distribution patterns of subsurface copepods and the impact of environmental parameters. Limnologica 42: 156-164.

Meleg, I. N., O. T. Moldovan, S. Iepure, F. Fiers & T. Brad, 2011. Diversity patterns of fauna in dripping water of caves from Transylvania. Annales De Limnologie-International Journal of Limnology 47: 185-197.

Meleg, I. N., M. Naparus, F. Fiers, I. H. Meleg, M. Vlaicu & O. T. Moldovan, 2014. The relationships between land cover, climate and cave copepod spatial distribution and suitability along the Carpathians. Environmental Conservation 41: 206-216.

Mencio, A., K. L. Korbel & G. C. Hose, 2014. River-aquifer interactions and their relationship to stygofauna assemblages: A case study of the Gwydir River alluvial aquifer (New South Wales, Australia). Science of the Total Environment 479: 292-305.

Mermillod-Blondin, F., M. C. Des Chatelliers, P. Marmonier & M. J. Dole-Olivier, 2000. Distribution of solutes, microbes and invertebrates in river sediments along a riffle-pool-riffle sequence. Freshwater Biology 44: 255-269.

Mermillod-Blondin, F., T. Winiarski, A. Foulquier, A. Perrissin & P. Marmonier, 2015. Links between sediment structures and ecological processes in the hyporheic zone: ground-penetrating radar as a non-invasive tool to detect subsurface biologically active zones. Ecohydrology 8: 626-641.

Michel, G., F. Malard, L. Deharveng, T. Di Lorenzo, B. Sket & C. De Broyer, 2009. Reserve selection for conserving groundwater biodiversity. Freshwater Biology 54: 861-876.

Milanovic, P., 2002. The environmental impacts of human activities and engineering constructions in karst regions. Episodes 25: 13-21.

Mokany, K., T. D. Harwood, S. A. Halse & S. Ferrier, 2019. Riddles in the dark: Assessing diversity patterns for cryptic subterranean fauna of the Pilbara. Diversity and Distributions 25: 240-254.

Moldovan, O. T., S. Constantin & S. Cheval, 2018. Drip heterogeneity and the impact of decreased flow rates on the vadose zone fauna in Ciur-Izbuc Cave, NW Romania. Ecohydrology 11.

Moldovan, O. T. & E. A. Levei, 2015. Temporal variability of fauna and the importance of sampling frequency in the hyporheic zone. Hydrobiologia 755: 27-38.

Moldovan, O. T., E. A. Levei, C. Marin, M. Banciu, H. L. Banciu, C. Pavelescu, T. Brad, M. Cimpean, I. Meleg, S. Iepure & I. Povara, 2011. Spatial distribution patterns of the hyporheic invertebrate communities in a polluted river in Romania. Hydrobiologia 669: 63-82.

Moldovan, O. T., I. N. Meleg, E. A. Levei & M. Terente, 2013. A simple method for assessing biotic indicators and predicting biodiversity in the hyporheic zone of a river polluted with metals. Ecological Indicators 24: 412-420.

Moldovan, O. T., I. N. Meleg & A. Persoiu, 2012. Habitat fragmentation and its effects on groundwater populations. Ecohydrology 5: 445-452.

Mori, N., T. Kanduc, M. Opalicki Slabe & A. Brancelj, 2015. Groundwater Drift as a Tracer for Identifying Sources of Spring Discharge. Groundwater 53: 123-132.

Moritsch, M. M., M. J. Pakes & D. R. Lindberg, 2014. How might sea level change affect arthropod biodiversity in anchialine caves: a comparison of Remipedia and Atyidae taxa (Arthropoda: Altocrustacea). Organisms Diversity & Evolution 14: 225-235.

Mosslacher, F., 1998. Subsurface dwelling crustaceans as indicators of hydrological conditions, oxygen concentrations, and sediment structure in an alluvial aquifer. International Review of Hydrobiology 83: 349-364.

Mugnai, R., G. Messana & T. Di Lorenzo, 2015. The hyporheic zone and its functions: revision and research status in Neotropical regions. Brazilian Journal of Biology 75: 524-534.

Murphy, N. P., M. Adams & A. D. Austin, 2009. Independent colonization and extensive cryptic speciation of freshwater amphipods in the isolated groundwater springs of Australia's Great Artesian Basin. Molecular Ecology 18: 109-122.

Murphy, N. P., M. T. Guzik & J. W. Wilmer, 2010. The influence of landscape on population structure of four invertebrates in groundwater springs. Freshwater Biology 55: 2499-2509.

Nakano, T. & K. Tomikawa, 2018. Reassessment of the Groundwater Amphipod Paramoera relicta Synonymizes the Genus Relictomoera with Paramoera (Crustacea: Amphipoda: Pontogeneiidae). Zoological Science 35: 459-467.

Nakano, T., K. Tomikawa & M. J. Grygier, 2018. Rediscovered syntypes of Procrangonyx japonicus, with nomenclatural consideration of some crangonyctoidean subterranean amphipods (Crustacea: Amphipoda: Allocrangonyctidae, Niphargidae, Pseudocrangonyctidae). Zootaxa 4532: 86-94.

Niemiller, M. L., G. O. Graening, D. B. Fenolio, J. C. Godwin, J. R. Cooley, W. D. Pearson, B. M. Fitzpatrick & T. J. Near, 2013. Doomed before they are described? The need for conservation assessments of cryptic species complexes using an amblyopsid cavefish (Amblyopsidae: Typhlichthys) as a case study. Biodiversity and Conservation 22: 1799-1820.

Nkemegni, G. N., S. H. Z. Togouet, A. Fomena, O. F. Pountougnigni & C. Piscart, 2015. Aquatic invertebrate fauna of wells in a tropical mountain climate, western Cameroon. African Journal of Aquatic Science 40: 393-401.

Notenboom, J., R. Serrano, I. Morell & F. Hernandez, 1995. The Phreatic Aquifer of the Plana De Castellon (Spain) - Relationships between Animal Assemblages and Groundwater Pollution. Hydrobiologia 297: 241-249.

Olafsdottir, J. H., J. G. Thorbjornsson, B. K. Kristjansson & J. S. Olafsson, 2019. Invertebrate biodiversity in cold groundwater fissures in Iceland. Ecology and Evolution 9: 6399-6409.

Olsen, D. A. & C. R. Townsend, 2003. Hyporheic community composition in a gravel-bed stream: influence of vertical hydrological exchange, sediment structure and physicochemistry. Freshwater Biology 48: 1363-1378.

Omesova, M. & J. Helesic, 2010. Organic matter and fine grains as possible determinants of spatial and seasonal variability in bed sediment fauna: A case study from a Hercynian gravel stream. Limnologica 40: 307-314.

Pacioglu, O. & O. T. Moldovan, 2016. Response of invertebrates from the hyporheic zone of chalk rivers to eutrophication and land use. Environmental Science and Pollution Research 23: 4729-4740.

Page, T. J., W. F. Humphreys & J. M. Hughes, 2008. Shrimps Down Under: Evolutionary Relationships of Subterranean Crustaceans from Western Australia (Decapoda: Atyidae: Stygiocaris). Plos One 3.

Pandourski, I. S. & M. Bobic, 2008. Acanthocyclops iskrecensis PANDOURSKI, 1992 (Crustacea, Copepoda, Cyclopoida) from the karstic groundwaters of Eastern Serbia. Acta Zoologica Bulgarica 60: 193-195.

Peralta, M., 2014. A new stygobitic species of Stygocarididae (Crustacea: Anaspidacea) from South America. Zootaxa 3760: 396-408.

Perina, G., A. I. Camacho, J. Huey, P. Horwitz & A. Koenders, 2018. Understanding subterranean variability: the first genus of Bathynellidae (Bathynellacea, Crustacea) from Western Australia described through a morphological and multigene approach. Invertebrate Systematics 32: 423-447.

Perina, G., A. I. Camacho, J. Huey, P. Horwitz & A. Koenders, 2019a. New Bathynellidae (Crustacea) taxa and their relationships in the Fortescue catchment aquifers of the Pilbara region, Western Australia. Systematics and Biodiversity 17: 148-164.

Perina, G., A. I. Camacho, J. Huey, P. Horwitz & A. Koenders, 2019b. The role of allopatric speciation and ancient origins of Bathynellidae (Crustacea) in the Pilbara (Western Australia): two new genera from the De Grey River catchment. Contributions to Zoology 88: 452-497.

Pesce, G. L., 1985. The Groundwater Fauna of Italy - a Synthesis. Stygologia 1: 129-159.

Peterson, J. W., E. M. Johnson, J. L. Cencer & C. J. Thomason, 2006. Physiochemical conditions of Folsomia candida occurrence in a shallow coastal Lake Michigan aquifer. Environmental Geology 49: 1125-1138.

Petts, G. E. & M. A. Bickerton, 1994. Influence of Water Abstraction on the Macroinvertebrate Community Gradient within a Glacial Stream System - La Borgne Darolla, Valais, Switzerland. Freshwater Biology 32: 375-386.

Pfister, G., J. Rieb, M. Avramov, T. Rock, C. Griebler & K. W. Schramm, 2013. Detection of catecholamines in single specimens of groundwater amphipods. Analytical and Bioanalytical Chemistry 405: 5571-5582.

Pinder, A. M., S. M. Eberhard & W. F. Humphreys, 2006. New phallodrilines (Annelida : Clitellata : Tubificidae) from Western Australian groundwater. Zootaxa: 31-48.

Pipan, T., C. Fiser, T. Novak & D. C. Culver, 2012. Fifty Years of the Hypotelminorheic: What Have We Learned? Acta Carsologica 41: 275-285.

Plenet, S., J. Gibert & P. Marmonier, 1995. Biotic and Abiotic Interactions between Surface and Interstitial Systems in Rivers. Ecography 18: 296-309.

Plenet, S., H. Hugueny & J. Gibert, 1996. Invertebrate community responses to physical and chemical factors at the river aquifer interaction zone .2. Downstream from the city of Lyon. Archiv Fur Hydrobiologie 136: 65-88.

Por, F. D., 2007. Ophel: a groundwater biome based on chemoautotrophic resources. The global significance of the Ayyalon cave finds, Israel. Hydrobiologia 592: 1-10.

Pospisil, P. & D. L. Danielopol, 2000. Diversity of groundwater dwelling cyclopoida (Crustacea, Copepoda) in a Danube Wetland in Austria. Vie Et Milieu-Life and Environment 50: 137-150.

Rabelo, L. M., M. Souza-Silva & R. L. Ferreira, 2018. Priority caves for biodiversity conservation in a key karst area of Brazil: comparing the applicability of cave conservation indices. Biodiversity and Conservation 27: 2097-2129.

Rasines-Ladero, R. & S. Iepure, 2016. Parent lithology and organic matter influence the hyporheic biota of two Mediterranean rivers in central Spain. Limnetica 35: 19-36.

Reboleira, A. S. P. S., N. Abrantes, P. Oromi & F. Goncalves, 2013. Acute Toxicity of Copper Sulfate and Potassium Dichromate on Stygobiont Proasellus: General Aspects of Groundwater Ecotoxicology and Future Perspectives. Water Air and Soil Pollution 224.

Reboleira, A. S. P. S., P. A. V. Borges, F. Goncalves, A. R. M. Serrano & P. Oromi, 2011. The subterranean fauna of a biodiversity hotspot region - Portugal: an overview and its conservation. International Journal of Speleology 40: 23-37.

Reddy, Y. R., 2018. An Updated Checklist of Groundwater Crustaceans of India. Crustaceana 91: 677-731.

Reddy, Y. R., V. R. Totakura & S. Shaik, 2016. A new genus and two new species of Parastenocarididae (Copepoda: Harpacticoida) from southeastern India. Journal of Natural History 50: 1315-1356.

Reeves, J. M., P. De Deckker & S. A. Halse, 2007. Groundwater Ostracods from the arid Pilbara region of northwestern Australia: distribution and water chemistry. Hydrobiologia 585: 99-118.

Ribera, I. & A. S. P. S. Reboleira, 2019. The first stygobiont species of Coleoptera from Portugal, with a molecular phylogeny of the Siettitia group of genera (Dytiscidae, Hydroporinae, Hydroporini, Siettitiina). Zookeys: 21-38.

Ring, R. A., 1991. The Insect Fauna and Some Other Characteristics of Natural Salt Springs on Saltspring Island, British-Columbia. Memoirs of the Entomological Society of Canada: 51-61.

Rios-Escalante, P. D. L., L. Parra-Coloma, M. A. Peralta, J. Perez-Schultheiss & E. H. Rudolph, 2016. A checklist of subterranean water crustaceans from Chile (South America). Proceedings of the Biological Society of Washington 129: 114-128.

Robertson, A. L., J. W. N. Smith, T. Johns & G. S. Proudlove, 2009. The distribution and diversity of stygobites in Great Britain: an analysis to inform groundwater management. Quarterly Journal of Engineering Geology and Hydrogeology 42: 359-368.

Rodriguez, P. & A. Achurra, 2010. New species of aquatic oligochaetes (Annelida: Clitellata) from groundwaters in karstic areas of northern Spain, with taxonomic remarks on Lophochaeta ignota Stolc, 1886. Zootaxa: 21-39.

Rogulj, B., P. Marmonier, R. Lattinger & D. Danielopol, 1994. Fine-Scale Distribution of Hypogean Ostracoda in the Interstitial Habitats of the Rivers Sava and Rhone. Hydrobiologia 287: 19-28.

Rossetti, G., V. Pieri & K. Martens, 2005. Recent ostracods (Crustacea, Ostracoda) found in lowland springs of the provinces of Piacenza and Parma (Northern Italy). Hydrobiologia 542: 287-296.

Rouch, R. & D. L. Danielopol, 1997. Species richness of microcrustacea in subterranean freshwater habitats. Comparative analysis and approximate evaluation. Internationale Revue Der Gesamten Hydrobiologie 82: 121-145.

Rouch, R., A. Mangin, M. Bakalowicz & D. DHulst, 1997. The hyporheic zone: Hydrogeological and geochemical study of a stream in the Pyrenees Mountains. Internationale Revue Der Gesamten Hydrobiologie 82: 357-378.

Ruffo, S. & R. Vonk, 2001. Ingolfiella beatricis, new species (Amphipoda : Ingolfiellidae) from subterranean waters of Slovenia. Journal of Crustacean Biology 21: 484-491.

Rundle, S. D., D. T. Bilton & D. K. Shiozawa, 2000. Global and regional patterns in lotic meiofauna. Freshwater Biology 44: 123-134.

Sacco, M., A. Blyth, P. W. Bateman, Q. Hua, D. Mazumder, W. Nicole, W. F. Humphreys, A. Laini, C. Griebler & K. Grice, 2019. New light in the dark - a proposed multidisciplinary framework for studying functional ecology of groundwater fauna. Science of the Total Environment 662: 963-977.

Sarkka, J., L. Levonen & J. Makela, 1998. Harpacticoid and cyclopoid fauna of groundwater and springs in southern Finland. Journal of Marine Systems 15: 155-161.

Sarkka, J. & J. Makela, 1999. Meiofauna of esker groundwaters in Finland. Hydrobiologia 405: 25-37.

Scarsbrook, M. R. & G. D. Fenwick, 2003. Preliminary assessment of crustacean distribution patterns in New Zealand groundwater aquifers. New Zealand Journal of Marine and Freshwater Research 37: 405-413.

Scarsbrook, M. R. & J. Halliday, 2002. Detecting patterns in hyporheic community structure: does sampling method alter the story? New Zealand Journal of Marine and Freshwater Research 36.

Schenkova, J., P. Paril, K. Petrivalska & J. Bojkova, 2010. Aquatic oligochaetes (Annelida: Clitellata) of the Czech Republic: check-list, new records, and ecological remarks. Zootaxa: 29-44.

Schmidt, S. I. & H. J. Hahn, 2012. What is groundwater and what does this mean to fauna? - An opinion. Limnologica 42: 1-6.

Schmidt, S. I., H. J. Hahn, T. J. Hatton & W. F. Humphreys, 2007a. Do faunal assemblages reflect the exchange intensity in groundwater zones? Hydrobiologia 583: 1-19.

Schmidt, S. I., H. J. Hahn, G. D. Watson, R. J. Woodbury & T. J. Hatton, 2004. Sampling fauna in stream sediments as well as groundwater using one net sampler. Acta Hydrochimica Et Hydrobiologica 32: 131-137.

Schmidt, S. I., J. Hellweg, H. J. Hahn, T. J. Hatton & W. F. Humphreys, 2007b. Does groundwater influence the sediment fauna beneath a small, sandy stream? Limnologica 37: 208-225.

Schminke, H. K., 2008. First Report of Groundwater Fauna from Papua New Guinea: Kinnecaris Jakobi, 1972 Redefined (Copepoda, Harpacticoida, Parastenocarididae), and Description of a New Species. Crustaceana 81: 1241-1253.

Schon, I., K. Martens & S. Halse, 2010. Genetic diversity in Australian ancient asexual Vestalenula (Ostracoda, Darwinulidae): little variability down under. Hydrobiologia 641: 59-70.

Shaik, S. & Y. R. Reddy, 2018. On the genus Habrobathynella Schminke, 1973 (Crustacea, Malacostraca, Bathynellacea), with description of three new species from India. Zootaxa 4492: 1-72.

Shapouri, M., L. C. da Fonseca, S. Iepure, T. Stigter, L. Ribeiro & A. Silva, 2016. The variation of stygofauna along a gradient of salinization in a coastal aquifer. Hydrology Research 47: 89-103.

Sidorov, D. A. & A. A. Gontcharov, 2013. Studies on subterranean amphipod crustaceans of Primory, Russia. Part 1. Three new species of the genus Pseudocrangonyx from springs and other groundwater habitats in far eastern Russia. Zootaxa 3693: 547-567.

Smith, H. & P. J. Wood, 2002. Flow permanence and macroinvertebrate community variability in limestone spring systems. Hydrobiologia 487: 45-58.

Sorensen, J. P. R., L. Maurice, F. K. Edwards, D. J. Lapworth, D. S. Read, D. Allen, A. S. Butcher, L. K. Newbold, B. R. Townsend & P. J. Williams, 2013. Using Boreholes as Windows into Groundwater Ecosystems. Plos One 8.

Stein, H., C. Griebler, S. Berkhoff, D. Matzke, A. Fuchs & H. J. Hahn, 2012. Stygoregions - a promising approach to a bioregional classification of groundwater systems. Scientific Reports 2.

Stein, H., C. Kellermann, S. I. Schmidt, H. Brielmann, C. Steube, S. E. Berkhoff, A. Fuchs, H. J. Hahn, B. Thulin & C. Griebler, 2010. The potential use of fauna and bacteria as ecological indicators for the assessment of groundwater quality. Journal of Environmental Monitoring 12: 242-254.

Stocchino, G. A., R. Sluys, M. Kawakatsu, S. M. Sarbu & R. Manconi, 2017. A new species of freshwater flatworm (Platyhelminthes, Tricladida, Dendrocoelidae) inhabiting a chemoautotrophic groundwater ecosystem in Romania. European Journal of Taxonomy 342: 1-21.

Stoch, F. & D. M. P. Galassi, 2010. Stygobiotic crustacean species richness: a question of numbers, a matter of scale. Hydrobiologia 653: 217-234.

Stock, J. H., 1994. Biogeographic Synthesis of the Insular Groundwater Faunas of the (Sub)Tropical Atlantic. Hydrobiologia 287: 105-117.

Strayer, D. L., S. E. May, P. Nielsen, W. Wollheim & S. Hausam, 1995. An Endemic Groundwater Fauna in Unglaciated Eastern North-America. Canadian Journal of Zoology-Revue Canadienne De Zoologie 73: 502-508.

Stumpp, C. & G. C. Hose, 2013. The Impact of Water Table Drawdown and Drying on Subterranean Aquatic Fauna in In-Vitro Experiments. Plos One 8.

Tang, D. & B. Knott, 2009. Freshwater cyclopoids and harpacticoids (Crustacea: Copepoda) from the Gnangara Mound region of Western Australia. Zootaxa: 1-70.

Tockner, K., F. Malard, P. Burgherr, C. T. Robinson, U. Uehlinger, R. Zah & J. V. Ward, 1997. Physico-chemical characterization of channel types in a glacial floodplain ecosystem (Val Roseg, Switzerland). Archiv Fur Hydrobiologie 140: 433-463.

Tomikawa, K. & T. Nakano, 2018. Two new subterranean species of Pseudocrangonyx Akatsuka & Komai, 1922 (Amphipoda: Crangonyctoidea: Pseudocrangonyctidae), with an insight into groundwater faunal relationships in western Japan. Journal of Crustacean Biology 38: 460-474.

Vanderkamp, G., 1995. The Hydrogeology of Springs in Relation to the Biodiversity of Spring Fauna - a Review. Journal of the Kansas Entomological Society 68: 4-17.

Victor, R. & A. A. I. Al-Farsi, 2001. Water quality and invertebrate fauna of farm wells in an area affected by salinization in Oman. Journal of Arid Environments 48: 419-428.

Villemant, C., C. Daugeron, O. Gargominy, M. Isaia, L. Deharveng & M. L. I. Judson, 2015. The Mercantour/Alpi Marittime All Taxa Biodiversity Inventory (ATBI): achievements and prospects. Zoosystema 37: 667-679.

von Fumetti, S., F. Bieri-Wigger & P. Nagel, 2017. Temperature variability and its influence on macroinvertebrate assemblages of alpine springs. Ecohydrology 10.

Voros, J., O. Marton, B. R. Schmidt, J. T. Gal & D. Jelic, 2017. Surveying Europe's Only Cave-Dwelling Chordate Species (Proteus anguinus) Using Environmental DNA. Plos One 12.

Wagner, H. P., 2012. Tethysbaena Ophelicola N. Sp (Thermosbaenacea), a New Prime Consumer in the Ophel Biome of the Ayyalon Cave, Israel. Crustaceana 85: 1571-1587.

Ward, J. V., 1994. Ecology of Alpine Streams. Freshwater Biology 32: 277-294.

Ward, J. V., G. Bretschko, M. Brunke, D. Danielopol, J. Gibert, T. Gonser & A. G. Hildrew, 1998. The boundaries of river systems: the metazoan perspective. Freshwater Biology 40: 531-569.

Watts, C. H. S. & W. F. Humphreys, 2009. Fourteen New Dytiscidae (Coleoptera) of the Genera Limbodessus Guignot, Paroster Sharp, and Exocelina Broun from Underground Waters in Australia. Transactions of the Royal Society of South Australia 133: 62-107.

White, J. C., A. House, N. Punchard, D. M. Hannah, N. A. Wilding & P. J. Wood, 2018. Macroinvertebrate community responses to hydrological controls and groundwater abstraction effects across intermittent and perennial headwater streams. Science of the Total Environment 610: 1514-1526.

Williams, D. D., 2003. The brackishwater hyporheic zone: invertebrate community structure across a novel ecotone. Hydrobiologia 510: 153-173.

Williams, D. D., N. E. Williams & Y. Cao, 1997. Spatial differences in macroinvertebrate community structure in springs in southeastern Ontario in relation to their chemical and physical environments. Canadian Journal of Zoology-Revue Canadienne De Zoologie 75: 1404-1414.

Wilson, G. D. F., 2008. Gondwanan groundwater: subterranean connections of Australian phreatoicidean isopods (Crustacea) to India and New Zealand. Invertebrate Systematics 22: 301-310.

Wood, P. J. & P. D. Armitage, 2004. The response of the macroinvertebrate community to low-flow variability and supra-seasonal drought within a groundwater dominated stream. Archiv Fur Hydrobiologie 161: 1-20.

Wood, P. J., J. Gunn & J. Perkins, 2002. The impact of pollution on aquatic invertebrates within a subterranean ecosystem - out of sight out of mind. Archiv Fur Hydrobiologie 155: 223-237.

Wood, P. J., J. Gunn & S. D. Rundle, 2008. Response of benthic cave invertebrates to organic pollution events. Aquatic Conservation-Marine and Freshwater Ecosystems 18: 909-922.

Wood, P. J., J. Gunn, H. Smith & A. Abas-Kutty, 2005. Flow permanence and macroinvertebrate community diversity within groundwater dominated headwater streams and springs. Hydrobiologia 545: 55-64.

Yacoubi-Khebiza, M., N. Coineau, C. Boutin & F. De Bovee, 1999. Interstitial crustaceans and groundwater quality in five rivers of the western High Atlas (Morocco). Crustaceana 72: 893-898.

Yao, J. M., J. M. Sanchez-Perez, S. Sauvage, S. Teissier, E. Attard, B. Lauga, R. Duran, F. Julien, L. Bernard-Jannin, H. Ramburn & M. Gerino, 2017. Biodiversity and ecosystem purification service in an alluvial wetland. Ecological Engineering 103: 359-371.

Zagmajster, M., D. C. Culver, M. C. Christman & B. Sket, 2010. Evaluating the sampling bias in pattern of subterranean species richness: combining approaches. Biodiversity and Conservation 19: 3035-3048.

Zaksek, V., B. Sket, S. Gottstein, D. Franjevic & P. Trontelj, 2009. The limits of cryptic diversity in groundwater: phylogeography of the cave shrimp Troglocaris anophthalmus (Crustacea: Decapoda: Atyidae). Molecular Ecology 18: 931-946.

**References list for the research key “spring fauna freshwater”**

Abramova, E., I. Vishnyakova, J. Boike, A. Abramova, G. Solovyev & F. Martynov, 2017. Structure of freshwater zooplankton communities from tundra waterbodies in the Lena River Delta, Russian Arctic, with a discussion on new records of glacial relict copepods. Polar Biology 40: 1629-1643.

Albrecht, C., T. Hauffe, K. Schreiber, S. Trajanovski & T. Wilke, 2009. Mollusc Biodiversity and Endemism in the Potential Ancient Lake Trichonis, Greece. Malacologia 51: 357-375.

Alkalaj, J., T. Hrafnsdottir, F. Ingimarsson, R. J. Smith, A. K. Kreiling & S. Mischke, 2019. Distribution of Recent non-marine ostracods in Icelandic lakes, springs, and cave pools. Journal of Crustacean Biology 39: 202-212.

Baars, J. R., D. A. Murray, E. Hannigan & M. Kelly-Quinn, 2014. Macroinvertebrate Assemblages of Small Upland Peatland Lakes in Ireland. Biology and Environment-Proceedings of the Royal Irish Academy 114b: 233-248.

Barquin, J. & R. G. Death, 2004. Patterns of invertebrate diversity in streams and freshwater springs in Northern Spain. Archiv Fur Hydrobiologie 161: 329-349.

Barquin, J. & R. G. Death, 2011. Downstream changes in spring-fed stream invertebrate communities: the effect of increased temperature range? Journal of Limnology 70: 134-146.

Bergersen, R., 1995. Is Greenland a Zoogeographical Unit of Its Own. Journal of Biogeography 22: 1-6.

Bogan, M. T., N. Noriega-Felix, S. L. Vidal-Aguilar, L. T. Findley, D. A. Lytle, O. G. Gutierrez-Ruacho, J. A. Alvarado-Castro & A. Varela-Romero, 2014. Biogeography and conservation of aquatic fauna in spring-fed tropical canyons of the southern Sonoran Desert, Mexico. Biodiversity and Conservation 23: 2705-2748.

Boix, D., J. Sala, S. Gascon, M. Martinoy, J. Gifre, S. Brucet, A. Badosa, R. Lopez-Flores & X. D. Quintana, 2007. Comparative biodiversity of crustaceans and aquatic insects from various water body types in coastal Mediterranean wetlands. Hydrobiologia 584: 347-359.

Brancelj, A. & T. Karanovic, 2015. A new subterranean Maraenobiotus (Crustacea: Copepoda) from Slovenia challenges the concept of polymorphic and widely distributed harpacticoids. Journal of Natural History 49: 2905-2928.

Brim-Box, J., J. Davis, K. Strehlow, G. McBurnie, A. Duguid, C. Brock, K. McConnell, C. Day & C. Palmer, 2014. Persistence of central Australian aquatic invertebrate communities. Marine and Freshwater Research 65: 562-572.

Callanan, M., J. R. Baars & M. Kelly-Quinn, 2008. Critical influence of seasonal sampling on the ecological quality assessment of small headwater streams. Hydrobiologia 610: 245-255.

Castro, B. B., S. M. Marques & F. Goncalves, 2007. Habitat selection and diel distribution of the crustacean zooplankton from a shallow Mediterranean lake during the turbid and clear water phases. Freshwater Biology 52: 421-433.

Colgan, D. J. & W. F. Ponder, 1994. The Evolutionary Consequences of Restrictions on Gene Flow - Examples from Hydrobiid Snails. Nautilus 108: 25-43.

Collado, G. A. & M. A. Mendez, 2013. Microgeographic differentiation among closely related species of Biomphalaria (Gastropoda: Planorbidae) from the Andean Altiplano. Zoological Journal of the Linnean Society 169: 640-652.

Collier, K. J. & B. J. Smith, 2006. Distinctive invertebrate assemblages in rockface seepages enhance lotic biodiversity in northern New Zealand. Biodiversity and Conservation 15: 3591-3616.

Copilas-Ciocianu, D., M. Grabowski, L. Parvulescu & A. Petrusek, 2014. Zoogeography of epigean freshwater Amphipoda (Crustacea) in Romania: fragmented distributions and wide altitudinal variability. Zootaxa 3893: 243-260.

Coulson, S. J., P. Convey, K. Aakra, L. Aarvik, M. L. Avila-Jimenez, A. Babenko, E. M. Biersma, S. Bostrom, J. E. Brittain, A. M. Carlsson, K. Christoffersen, W. H. De Smet, T. Ekrem, A. Fjellberg, L. Fureder, D. Gustafsson, D. J. Gwiazdowicz, L. O. Hansen, M. Holmstrup, M. Hulle, L. Kaczmarek, M. Kolicka, V. Kuklinr, H. K. Lakka, N. Lebedeva, O. Makarova, K. Maraldo, E. Melekhina, F. Odegaard, H. E. Pilskog, J. C. Simon, B. Sohlenius, T. Solhoy, G. Soli, E. Stur, A. Tanasevitch, A. Taskaeva, G. Velle, K. Zawierucha & K. Zmudczynska-Skarbek, 2014. The terrestrial and freshwater invertebrate biodiversity of the archipelagoes of the Barents Sea, Svalbard, Franz Josef Land and Novaya Zemlya. Soil Biology & Biochemistry 68: 440-470.

Denisenko, N. V., S. G. Denisenko & K. K. Lehtonen, 2019. Distribution of macrozoobenthos in an Arctic estuary (Pechora Bay, SE Barents Sea) during the spring flood period. Polar Biology 42: 1667-1684.

Di Sabatino, A., B. Cicolani & R. Gerecke, 2003. Biodiversity and distribution of water mites (Acari, Hydrachnidia) in spring habitats. Freshwater Biology 48: 2163-2173.

Di Sabatino, A., R. Gerecke & P. Martin, 2000. The biology and ecology of lotic water mites (Hydrachnidia). Freshwater Biology 44: 47-62.

Di Sabatino, A., H. Smit, R. Gerecke, T. Goldschmidt, N. Matsumoto & B. Cicolani, 2008. Global diversity of water mites (Acari, Hydrachnidia; Arachnida) in freshwater. Hydrobiologia 595: 303-315.

Evtimova, V. V. & I. S. Pandourski, 2016. Rotifers and lower crustaceans from South-western Iceland. Biodiversity Data Journal 4.

Fjellheim, A. & G. G. Raddum, 2001. Acidification and liming of River Vikedal, western Norway. A 20 year study of responses in the benthic invertebrate fauna. Water Air and Soil Pollution 130: 1379-1384.

Friberg, N., A. Rebsdorf & S. E. Larsen, 1998. Effects of afforestation on acidity and invertebrates in Danish streams and implications for freshwater communities in Denmark. Water Air and Soil Pollution 101: 235-256.

Fureder, L., 2007. Life at the edge: Habitat condition and bottom fauna of Alpine running waters. International Review of Hydrobiology 92: 491-513.

Gribsholt, B., E. Struyf, A. Tramper, L. De Brabandere, N. Brion, S. van Damme, P. Meire, F. Dehairs, J. J. Middelburg & H. T. S. Boschker, 2007. Nitrogen assimilation and short term retention in a nutrient-rich tidal freshwater marsh - a whole ecosystem N-15 enrichment study. Biogeosciences 4: 11-26.

Gunn, J., P. Hardwick & P. J. Wood, 2000. The invertebrate community of the Peak-Speedwell cave system, Derbyshire, England pressures and considerations for conservation management. Aquatic Conservation-Marine and Freshwater Ecosystems 10: 353-369.

Gurlek, M. E., 2019. A new genus and two new species of freshwater Gastropoda from the Ceyhan River Basin in the eastern Mediterranean (Mollusca: Gastropoda: Truncatelloidea). Zoology in the Middle East 65: 59-65.

Humphreys, W. F., 2014. Subterranean fauna of Christmas Island: habitats and salient features. Raffles Bulletin of Zoology: 29-44.

Ilmonen, J., L. Paasivirta, R. Virtanen & T. Muotka, 2009. Regional and local drivers of macroinvertebrate assemblages in boreal springs. Journal of Biogeography 36: 822-834.

Jaume, D., 2008. Global diversity of spelaeogriphaceans & thermosbaenaceans (Crustacea; Spelaeogriphacea & Thermosbaenacea) in freshwater. Hydrobiologia 595: 219-224.

Johnson, P. D., A. E. Bogan, K. M. Brown, N. M. Burkhead, J. R. Cordeiro, J. T. Garner, P. D. Hartfield, D. A. W. Lepitzki, G. L. Mackie, E. Pip, T. A. Tarpley, J. S. Tiemann, N. V. Whelan & E. E. Strong, 2013. Conservation Status of Freshwater Gastropods of Canada and the United States. Fisheries 38: 247-282.

Kapakos, Y. & I. Karaouzas, 2019. Contribution to the knowledge of Atyidae and Palaemonidae (Crustacea: Decapoda) of Greece. Zootaxa 4543: 145-150.

Khatami, S. H. & S. J. Shayegan, 2006. Physico-chemical and biological characteristics of Sarabs (spring pools) in the Kermanshah Province of Iran. Iranian Journal of Science and Technology Transaction B-Engineering 30: 621-635.

Konopacka, A., K. Hupalo, T. Rewicz & M. Grabowski, 2014. Species inventory and distribution patterns of freshwater amphipods in Moldova. North-Western Journal of Zoology 10: 382-392.

Kulkoyluoglu, O., 2004. On the usage of ostracods (Crustacea) as bioindicator species in different aquatic habitats in the Bolu region, Turkey. Ecological Indicators 4: 139-147.

Kulkoyluoglu, O., 2009. Ecological Succession of Freshwater Ostracoda (Crustacea) in A Newly Developed Rheocrene Spring (Bolu, Turkey). Turkish Journal of Zoology 33: 115-123.

Kulkoyluoglu, O., M. Dugel & M. Kilic, 2007. Ecological requirements of Ostracoda (Crustacea) in a heavily polluted shallow lake, Lake Yenicaga (Bolu, Turkey). Hydrobiologia 585: 119-133.

Kulkoyluoglu, O. & G. L. Vinyard, 2000. Distribution and ecology of freshwater Ostracoda (Crustacea) collected from springs of Nevada, Idaho, and Oregon: A preliminary study. Western North American Naturalist 60: 291-303.

Lorenschat, J. & A. Schwalb, 2013. Autecology of the extant ostracod fauna of Lake Ohrid and adjacent waters - a key to paleoenvironmental reconstruction. Belgian Journal of Zoology 143: 42-68.

Manenti, R., 2014. Role of Cave Features for Aquatic Troglobiont Fauna Occurrence: Effects on "Accidentals" and Troglomorphic Organisms Distribution. Acta Zoologica Academiae Scientiarum Hungaricae 60: 257-270.

Martin, P., 2006. On the morphology and classification of larval water mites (Hydrachnidia, Acari) from springs in Luxembourg. Zootaxa: 1-44.

Martin, P., R. M. Schmelz & M. J. Dole-Olivier, 2015. Groundwater oligochaetes (Annelida, Clitellata) from the Mercantour National Park (France), with the descriptions of one new genus and two new stygobiont species. Zoosystema 37: 551-569.

Morales, E. S., M. A. Aguirre & E. J. Walsh, 2010. Freshwater Copepoda (Crustacea) from the Chihuahuan Desert with Comments on Biogeography. Southwestern Naturalist 55: 525-531.

Mori, N. & A. Brancelj, 2006. Macroinvertebrate communities of karst springs of two river catchments in the Southern Limestone Alps (the Julian Alps, NW Slovenia). Aquatic Ecology 40: 69-83.

Murphy, N. P., M. Adams & A. D. Austin, 2009. Independent colonization and extensive cryptic speciation of freshwater amphipods in the isolated groundwater springs of Australia's Great Artesian Basin. Molecular Ecology 18: 109-122.

Novichkova, A., E. Chertoprud & G. M. Gislason, 2014. Freshwater Crustacea (Cladocera, Copepoda) of Iceland: taxonomy, ecology, and biogeography. Polar Biology 37: 1755-1767.

Percin-Pacal, F., S. Altinsacli & S. Altinsacli, 2017. Distribution, diversity and some ecological characteristics of ostracods (Crustacea: Ostracoda) in Gokceada (Imbros) Island (Northern Aegean Sea, Turkey). Biologia 72: 774-789.

Perez, K. E., W. F. Ponder, D. J. Colgan, S. A. Clark & C. Lydeard, 2005. Molecular phylogeny and biogeography of spring-associated hydrobild snails of the Great Artesian Basin, Australia. Molecular Phylogenetics and Evolution 34: 545-556.

Pieri, V., C. Caserini, S. Gomarasca, K. Martens & G. Rossetti, 2007. Water quality and diversity of the Recent ostracod fauna in lowland springs from Lombardy (northern Italy). Hydrobiologia 585: 79-87.

Polaskova, V., J. Schenkova, M. Bartosova, V. Radkova & M. Horsak, 2017. Post-mining calcareous seepages as surrogate habitats for aquatic macroinvertebrate biota of vanishing calcareous spring fens. Ecological Engineering 109: 119-132.

Pozojevic, I., A. Brigic & S. Gottstein, 2018. Water mite (Acari: Hydrachnidia) diversity and distribution in undisturbed Dinaric karst springs. Experimental and Applied Acarology 76: 123-138.

Prejs, K., 1993. Distribution and Feeding of the Predatory Nematode Anatonchus-Dolichurus (Mononchoidea) in the Dokka Delta (Norway) and Its Impact on the Benthic Meiofauna. Freshwater Biology 29: 71-78.

Reiss, M., P. Martin, R. Gerecke & S. von Fumetti, 2016. Limno-ecological characteristics and distribution patterns of spring habitats and invertebrates from the Lowlands to the Alps. Environmental Earth Sciences 75.

Richards, R. R., K. K. Gates & B. L. Kerans, 2014. Effects of Simulated Rapid Water Level Fluctuations (Hydropeaking) on Survival of Sensitive Benthic Species. River Research and Applications 30: 954-963.

Robson, B. J. & C. J. Clay, 2005. Local and regional macroinvertebrate diversity in the wetlands of a cleared agricultural landscape in south-western Victoria, Australia. Aquatic Conservation-Marine and Freshwater Ecosystems 15: 403-414.

Rossetti, G., M. Bartoli & K. Martens, 2004. Limnological characteristics and recent ostracods (Crustacea, Ostracoda) of freshwater wetlands in the Parco Oglio Sud (Northern Italy). Annales De Limnologie-International Journal of Limnology 40: 329-341.

Rossetti, G., V. Pieri & K. Martens, 2005. Recent ostracods (Crustacea, Ostracoda) found in lowland springs of the provinces of Piacenza and Parma (Northern Italy). Hydrobiologia 542: 287-296.

Sari, N. & O. Kulkoyluoglu, 2010. Ostracods (Crustacea) and habitat similarities in the Bolu region (Turkey). Turkish Journal of Zoology 34: 225-230.

Savatenalinton, S. & M. Suttajit, 2016. A checklist of Recent non-marine ostracods (Crustacea: Ostracoda) from Thailand, including descriptions of two new species. Zootaxa 4067: 1-34.

Schmidlin, L., S. von Fumetti & P. Nagel, 2015a. Copper sulphate reduces the metabolic activity of Gammarus fossarum in laboratory and field experiments. Aquatic Toxicology 161: 138-145.

Schmidlin, L., S. von Fumetti & P. Nagel, 2015b. Effects of increased temperatures on Gammarus fossarum under the influence of copper sulphate. Ecotoxicology 24: 433-444.

Sirbu, I. & A. M. Benedek, 2017. Native Relict versus Alien Molluscs in Thermal Waters of North-Western Romania, Including the First Record of Melanoides tuberculata (O. F. Muller, 1774) (Gastropoda: Thiaridae) from Romania. Acta Zoologica Bulgarica 69: 31-36.

Spyra, A., 2017. Acidic, neutral and alkaline forest ponds as a landscape element affecting the biodiversity of freshwater snails. Science of Nature 104.

Strachan, S. R., E. T. Chester & B. J. Robson, 2016. Habitat alters the effect of false starts on seasonal-wetland invertebrates. Freshwater Biology 61: 680-692.

Strong, E. E., O. Gargominy, W. F. Ponder & P. Bouchet, 2008. Global diversity of gastropods (Gastropoda; Mollusca) in freshwater. Hydrobiologia 595: 149-166.

Suarez-Morales, E., F. Mendoza & N. Mercado-Salas, 2010. A new Allocyclops (Crustacea, Copepoda, Cyclopoida) from bromeliads and records of freshwater copepods from Mexico. Zoosystema 32: 393-407.

Tang, D. & B. Knott, 2009. Freshwater cyclopoids and harpacticoids (Crustacea: Copepoda) from the Gnangara Mound region of Western Australia. Zootaxa: 1-70.

Ucak, S., O. Kulkoyluoglu, D. Akdemir & E. Basak, 2014. Distribution, Diversity and Ecological Characteristics of Freshwater Ostracoda (Crustacea) in Shallow Aquatic Bodies of the Ankara Region, Turkey. Wetlands 34: 309-324.

Von Fumetti, S. & P. Nagel, 2011. A first approach to a faunistic crenon typology based on functional feeding groups. Journal of Limnology 70: 147-154.

von Fumetti, S., P. Nagel & B. Baltes, 2007. Where a springhead becomes a springbrook - a regional zonation of springs. Fundamental and Applied Limnology 169: 37-48.

Von Fumetti, S., P. Nagel, N. Scheifhacken & B. Baltes, 2006. Factors governing macrozoobenthic assemblages in perennial springs in north-western Switzerland. Hydrobiologia 568: 467-475.

Witt, J. D. S., D. W. Blinn & P. D. N. Hebert, 2003. The recent evolutionary origin of the phenotypically novel amphipod Hyalella montezuma offers an ecological explanation for morphological stasis in a closely allied species complex. Molecular Ecology 12: 405-413.

Worsham, M. L. D., R. Gibson & D. G. Huffman, 2016. The aquatic annelid fauna of the San Marcos River headsprings, Hays County, Texas. Zookeys: 1-14.

Yilmaz, F. & O. Kulkoyluoglu, 2006. Tolerance, optimum ranges, and ecological requirements of freshwater Ostracoda (Crustacea) in Lake Aladag (Bolu, Turkey). Ecological Research 21: 165-173.

Zganec, K., P. Lunko, A. Stroj, T. Mamos & M. Grabowski, 2016. Distribution, ecology and conservation status of two endemic amphipods, Echinogammarus acarinatus and Fontogammarus dalmatinus, from the Dinaric karst rivers, Balkan Peninsula. Annales De Limnologie-International Journal of Limnology 52: 13-26.
